# Deliberate practice of diagnostic clinical reasoning reveals low performance and improvement of diagnostic justification in pre-clerkship students

**DOI:** 10.1186/s12909-023-04541-5

**Published:** 2023-09-21

**Authors:** Justine Staal, Jason Waechter, Jon Allen, Chel Hee Lee, Laura Zwaan

**Affiliations:** 1 Erasmus Medical Center, Institute of Medical Education Research Rotterdam, Rotterdam, the Netherlands; 2 Department of Critical Care, Cumming School of Medicine, University of Calgary, Calgary, AB Canada; 3 Department of Medicine, University of North Dakota School of Medicine and Health Sciences, Grand Forks, ND USA; 4 Department of Mathematics, University of Calgary, Calgary, AB Canada; 5 Institute of Medical Education Research, Erasmus Medical Center, Dr. Molewaterplein 40, Rotterdam, 3015 GD the Netherlands

**Keywords:** Clinical reasoning, Assessment, Formative feedback, Curriculum, Deliberate practice

## Abstract

**Purpose:**

Diagnostic errors are a large burden on patient safety and improving clinical reasoning (CR) education could contribute to reducing these errors. To this end, calls have been made to implement CR training as early as the first year of medical school. However, much is still unknown about pre-clerkship students’ reasoning processes. The current study aimed to observe how pre-clerkship students use clinical information during the diagnostic process.

**Methods:**

In a prospective observational study, pre-clerkship medical students completed 10–11 self-directed online simulated CR diagnostic cases. CR skills assessed included: creation of the differential diagnosis (Ddx), diagnostic justification (DxJ), ordering investigations, and identifying the most probable diagnosis. Student performances were compared to expert-created scorecards and students received detailed individualized formative feedback for every case.

**Results:**

121 of 133 (91%) first- and second-year medical students consented to the research project. Students scored much lower for DxJ compared to scores obtained for creation of the Ddx, ordering tests, and identifying the correct diagnosis, (30–48% lower, p < 0.001). Specifically, students underutilized physical exam data (p < 0.001) and underutilized data that decreased the probability of incorrect diagnoses (p < 0.001). We observed that DxJ scores increased 40% after 10–11 practice cases (p < 0.001).

**Conclusions:**

We implemented deliberate practice with formative feedback for CR starting in the first year of medical school. Students underperformed in DxJ, particularly with analyzing the physical exam data and pertinent negative data. We observed significant improvement in DxJ performance with increased practice.

**Supplementary Information:**

The online version contains supplementary material available at 10.1186/s12909-023-04541-5.

## Introduction

Diagnostic errors, defined as missed, wrong, or delayed diagnoses, pose a significant burden on patient safety: most patients will likely experience one during their lifetime, sometimes with devastating consequences [1]. Flaws in clinical reasoning (CR), such as cognitive errors [2–8] or knowledge deficits, [9–12] are thought to be the main causes of diagnostic error [10, 13]. CR encompasses many complex cognitive skills [14, 15] and is a core competency for graduating medical students [16]. Therefore, improving CR training could contribute to reducing diagnostic errors [2, 13, 16, 17].

While *teaching* students about CR starts early in medical school, practice opportunities focused on *training* CR skill development does not start until clerkship, typically via observing expert clinicians and performing assessments on real patients [18, 19]. It is generally expected that students’ CR skills will improve markedly and sufficiently during clerkships; however, this is contradicted by research showing that students’ improvements are about similar to, or even less than their improvements in the pre-clerkship years [20]. This indicates that current CR training remains suboptimal, likely due to limitations in the methods for training and assessment of CR skills throughout medical school [17, 27]. Outside the workplace, both training and assessment are restricted by the time, funding, and manpower resources required to collect and analyze CR relevant data. Additionally, the current methods of assessment, such as using students’ final diagnostic accuracy, have been doubted in their sensitivity to truly measure CR [21].

One proposed solution includes beginning CR training for pre-clerkship students in first year medical school and throughout all phases of undergraduate medical education [1, 19, 22−27]. This will increase opportunities for formative feedback and allow students to start developing diagnostic skills prior to clinical rotations. Deliberate practice, the iterative process of repeated practicing and receiving formative feedback with simulation has also been proposed as an effective strategy for training CR [23, 25, 26]. Key aspects of CR that should be incorporated into medical school training and assessment include: building a Ddx, ordering tests, choosing a most probable diagnosis, and importantly, diagnostic justification [22, 25−27].

Diagnostic justification (DxJ) is the process of identifying clinical data that increases or decreases the probability that a diagnosis is the correct diagnosis (or alternatively, is not the correct diagnosis). DxJ performance was observed to be below expectations in medical students and differentiates experts from novices [25, 26, 29−31]. Novices made errors because they had difficulty recognizing or interpreting relevant information, [32, 33] had limited knowledge of pertinent information [33] and underreported both positive, and to a larger extent, negative pertinent information [34–36]. These findings have primarily been observed in medical students during or after their clerkship training and much remains unknown about the reasoning processes of pre-clerkship students. When included as a component of assessment, DxJ was found to be the most predictive of graduate competency exam performance, have the highest item discrimination and increased assessment reliability [26].

The current study aimed to determine how pre-clerkship medical students utilized clinical information in diagnostic cases. Our research questions (RQ) focused on overall processes of CR:

1. How do students perform at: creating a Ddx, performing DxJ, ordering and using investigations, and determining the correct Dx, and does this performance change with increased practice?
2. Within DxJ, are there differences in scores among the different categories of data (history, physical exam, and investigation results)?
3. Within DxJ, how do students assign data as increases versus decreases probability to the diagnoses in their Ddx and does this change with correct versus incorrect diagnoses?
4. How do first year students perform on all research questions compared to second year students?

We expected students would improve scores for all CR skills using deliberate practice (RQ1). RQ2 was observational and our null hypothesis was that there would be no differences. For RQ3 we expected more data to be assigned as “increases” than “decreases” for all diagnoses but we hypothesized that more data would be assigned to “decreases probability” for incorrect diagnoses than for correct diagnoses. Finally, we hypothesized that second year students would outperform first year students on all research questions (RQ4).

## Methods

This was a prospective single-site observational study, approved by the Conjoint Health Research Ethics Board at the University of Calgary (REB19-0065) and the University of North Dakota (IRB00001300).

### Participants

First and second year medical students were recruited from the University of North Dakota; the average age of the first-year medical students was 24. Simulated CR cases on teachingmedicine.com/dx were integrated in the mandatory curriculum and 133 students completed multiple cases throughout the school year. Students provided informed consent for their data to be included in research and did not receive compensation for participating.

### Case creation

Eleven text-based case vignettes were created for training. A 7-member committee representing internal medicine, critical care, neurology, cardiology, obstetrics, general surgery, and paediatrics brainstormed a minimum of three common diagnoses from 15 different systems/categories (total 58 diagnoses); 11 cases with typical presentations were created based on diagnoses from this list. The order of case presentation targeted 25% overlap with the currently taught organ system and 75% overlap with previously taught organ systems.

Each clinical scenario contained four stages: a one sentence introduction, the history, the physical exam, and investigations. The student could create a Ddx of up to 5 diagnoses and assign data from the history and physical exam to each diagnosis, indicating whether this data increases or decreases the probability of the diagnosis being correct. Students could do the same with investigation results. No investigation results were provided unless ordered by the student. Students could navigate back and forth between stages as needed. Further details about the CR software are described in a recent Innovation Report [37].

### Case completion by student

Students registered an online account on teachingmedicine.com, for which they provided their name, email, and a password. Cases were provided one at a time for the first-year students and one or two at a time for the second-year students. First year students completed 11 cases, one case per month and started the first case in the first month of their first year; second year students completed 10 cases spaced out over six months and started early in the second year. All students were given two to four weeks to complete each case and completed them during self-study. Students were encouraged to work in groups and to use internet searches and textbooks as needed. Case order was different between 1st and 2nd year students, but was the same for all students in a given year.

Students were provided with individualized formative feedback for each completed case. This feedback was based on a comparison between the over 100 data points collected per case and the scorecard (see *Scoring* below). Students’ iterative cycle of practice and feedback with each case comprises deliberate practice. The feedback provided both quantitative and qualitative information on the correct and incorrect choices they made when building their differential diagnosis, performing DxJ, ordering and using the results of investigations, and identifying the most probable diagnosis at the end of the case.

Students also attended a whole-class 1 hour video-conference review of each case, during which a faculty member demonstrated a “think aloud” demonstration of navigating the case, followed by a review of whole-class performance statistics and an informal online survey of the students. The survey results were collected and displayed to students and faculty during the review session and were used for curriculum improvement but were not included for research analysis.

### Scoring

A scorecard was created for each clinical scenario (see Supplemental Material A for example). Specific diagnoses were designated as “appropriate” for the case; if an appropriate diagnosis was added to the Ddx by the student, a point was earned for building their Ddx. Data from the history, physical exam, and investigations were coded as “required”, “neutral”, or “wrong” for each category of “increases” or “decreases” probability for every Dx submitted by all students. A point was: earned if data was assigned where “required”, missed if not assigned where “required” and half point deducted if assigned where “wrong”. Investigations were coded as “required” or “inappropriate”: the learner earned points for ordering appropriate tests and lost points for inappropriate tests. If the correct diagnosis was chosen as the most probable Dx for the case, the user scored 100% for this section. There were algorithms to score partial marks if the user did not assign the most probable Dx for the case as most probable, but instead, assigned it as less probable. Scores out of 10 were calculated for each of: Ddx, DxJ, investigations, and final diagnosis.

### Statistical analysis

Performance data and scoring were collected and calculated using teachingmedicine.com; the data were then de-identified, exported, and analyzed using the programming language and statistical environment R-4.0.1 (R Core Team). The Kruskal-Wallis test was used to compare: (1) students’ mean scores on creating a Ddx, performing DxJ, ordering and using investigations, and determining the most probable correct Dx; (2) how students performed on DxJ on the history, physical exam, and investigation results; and (3) how students assign pertinent positive and pertinent negative information to diagnoses. A linear mixed-effects model was used to explore changes in scores across cases. Finally, a Wilcoxon test and *t*-test were used to compare performance between first year and second year students.

## Results

121 of 133 (91%) first- and second-year medical students consented to the research project and completed 11 and 10 clinical cases respectively. All cases were completed between August 2021 and May 2022.

### RQ1: Student performance on building Ddx, DxJ, investigations, and final diagnosis

**Fig. 1:**
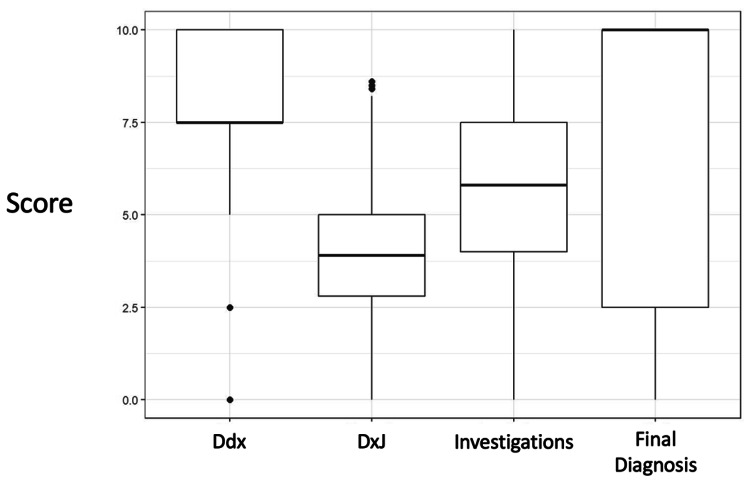
Mean performance of all students on the differential diagnosis, diagnostic justification, investigations, and final diagnosis Mean scores for building the differential diagnosis (Ddx), performing diagnostic justification (DxJ), ordering investigations, and identifying the correct diagnosis for all cases completed by all students. All scores are statistically different from each other (p < 0.001) with DxJ notably having the lowest score. The maximum score possible was 10 for each score. All scores are calculated independently from each other

**Fig. 2:**
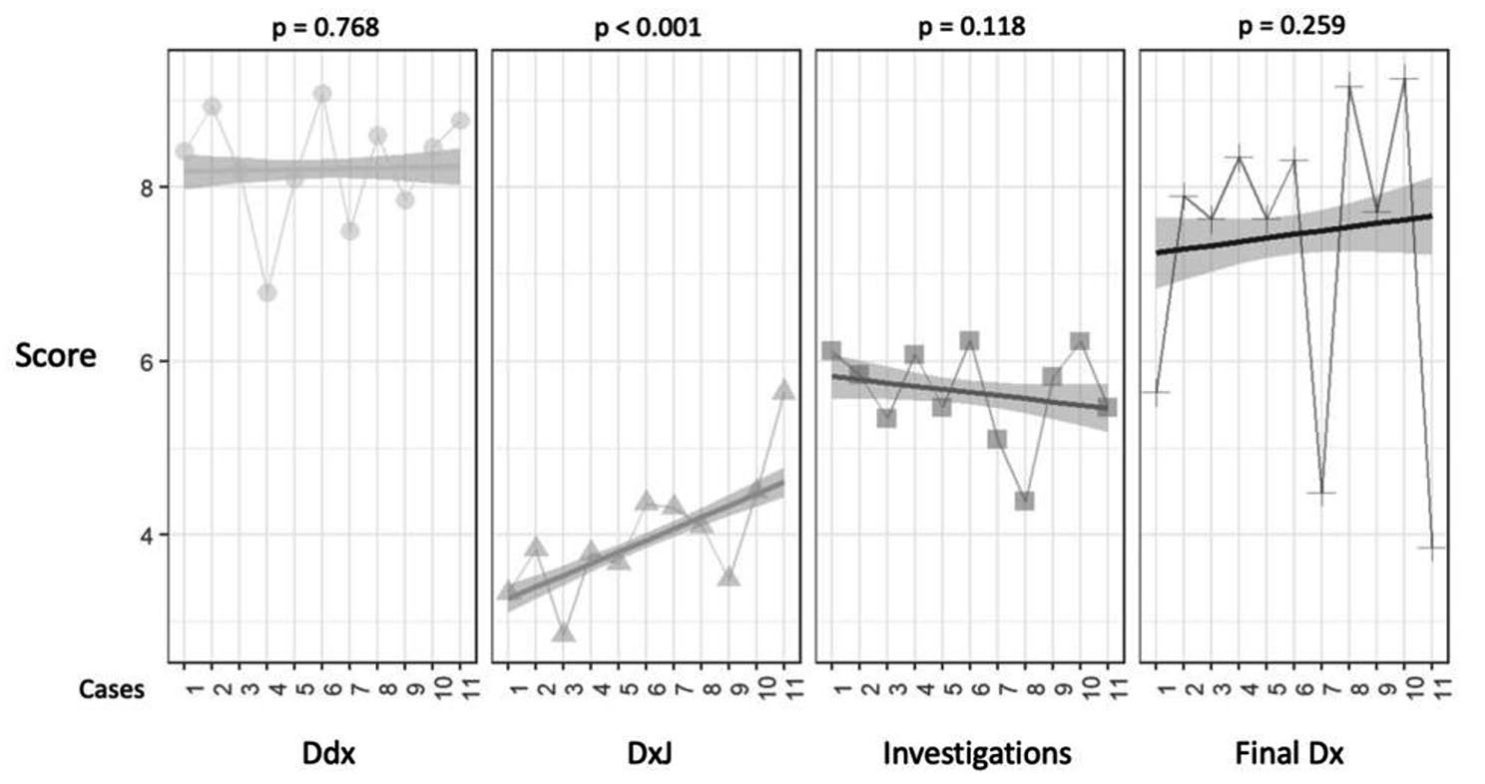
Changes in mean performance for all students per completed case The X axis shows completed cases 1 through 11 for both 1st year (11 cases) and 2nd year (10 cases) students; the Y axis shows the scores for each of: building the differential diagnosis (Ddx), diagnostic justification (DxJ), ordering investigations, and final diagnosis (Dx). Scores for DxJ increased by 40% (p < 0.001); no statistical changes were observed for the scores for Ddx, investigations, and final Dx across the cases

Figure 1 shows students’ mean scores across all cases and all students. Students’ mean scores differed significantly between building the Ddx (8.21, SD = 2.0), performing DxJ (3.9, SD = 1.6), investigations (5.65, SD = 2.6), and the final diagnosis (7.45, SD = 4.2) (*p* < 0.001). DxJ scores were 30% lower than scores for building the Ddx and 48% lower than for investigations. We investigated if these scores increased with deliberate practice (Fig. 2). Only DxJ scores increased over 10–11 cases, from 3.13 to 4.40, showing an increase of 40% improvement (*p* < 0.001).

### RQ4: first year versus second year student performance

**Fig. 3:**
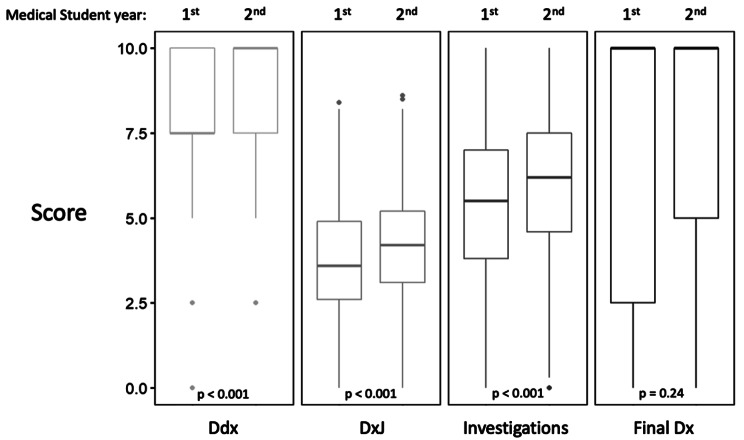
Mean performance of first year compared to second year students on the differential diagnosis, diagnostic justification, investigations, and final diagnosis Comparison of 1st and 2nd year students. There were statistical differences between 1st and 2nd year students for all scores except the final diagnosis (Dx) (p < 0.05)

Figure 3 shows that second year students scored higher than first year students for Ddx (7.88 vs. 8.56), DxJ (3.67 vs. 4.14), and investigations (5.39 vs. 5.92), (*p* < 0.001 for all) but not for final diagnosis (7.32 vs. 7.58, *p* = 0.24).

### RQ2: Student DxJ performance in history, physical exam, and investigations

**Fig. 4:**
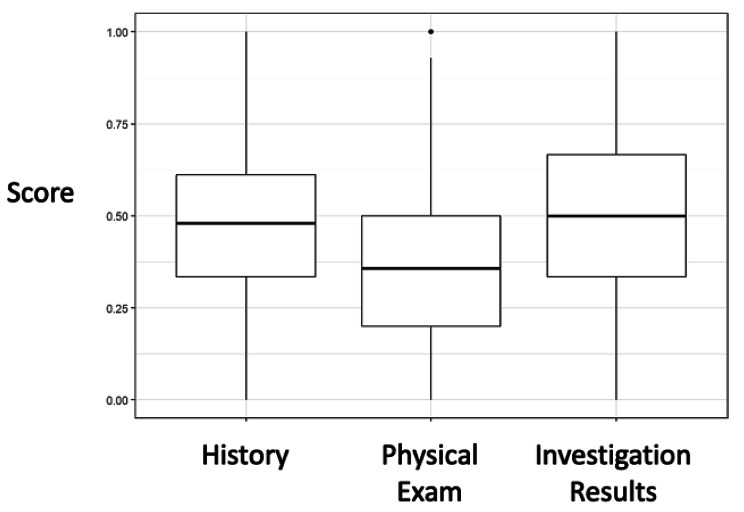
Mean performance for all students on diagnostic justification compared for the case history, physical exam, and investigations Mean diagnostic justification (DxJ) scores when classified by history, physical exam, and investigation results. Scores are scaled to a maximum of 1. Scores for the physical exam were significantly lower than both history and investigation results (p < 0.05)

Figure 4 displays students’ mean scores for DxJ in the history (M = 0.47, SD = 0.2), physical exam (M = 0.38, SD = 0.2), and investigations (M = 0.49, SD = 0.2). The maximum possible scaled value is 1.0. The physical exam score was significantly lower than the history and investigations scores. (*p* < 0.001); the difference between history and investigations was not statistically different (p = 0.058).

### RQ4: first year versus second year student performance

**Fig. 5:**
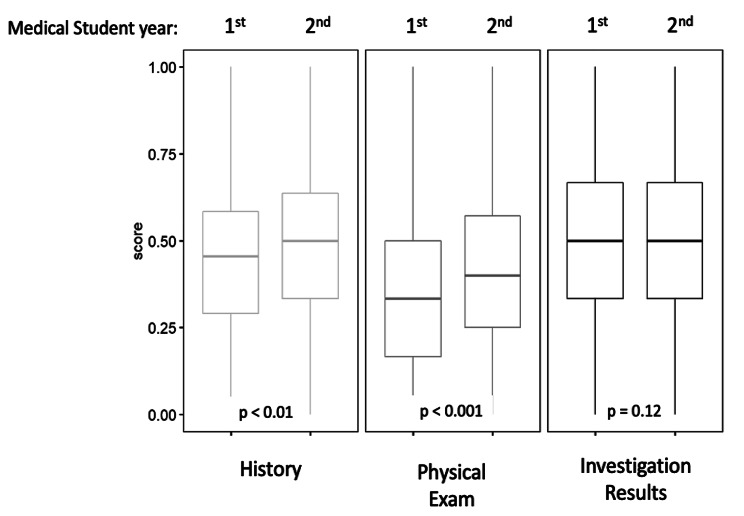
Mean performance for first year compared to second year students on diagnostic justification compared for the case history, physical exam, and investigations Comparison of scores for 1st versus 2nd year students. 2nd year students scored higher than 1st year students for diagnostic justification (DxJ) using history and physical exam data, but there was no observed difference for analyzing the investigation results

Figure 5 shows that second year students scored higher DxJ scores for history (*p* = 0.001) and physical exam (*p* < 0.001), but not for investigations (*p* = 0.12).

### RQ3: Student DxJ performance in assign data as increases versus decreases probability

**Fig. 6:**
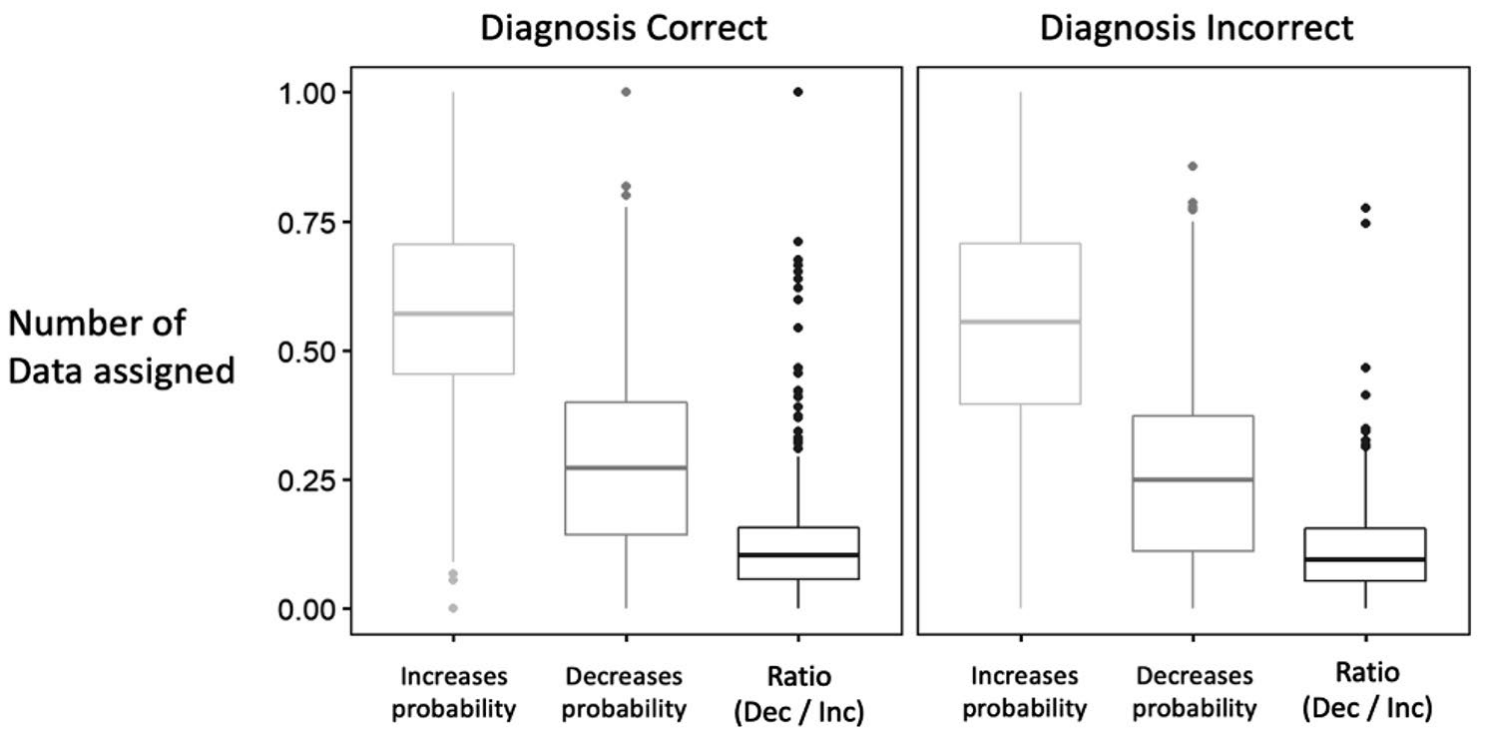
Mean performance of all students in assigning data increasing or decreasing the probability of a diagnosis during diagnostic justification The ratio of decreases to increases data, compared for correct diagnoses and incorrect diagnoses, was measured using the number of data assigned as “increases probability” and “decreases probability”. Data is not weighted with respect to importance or magnitude of impact on probabilities. We predicted and observed that data was much more frequently assigned as “increases” compared to “decreases” probability for both correct and incorrect diagnoses. We predicted the ratio of “decreases to increases” data to increase for incorrect diagnoses but we observed no statistical difference between the ratios (value = 0.13 for both, p = 0.41)

Figure 6 shows that students assigned significantly more data to the “increase probability” category than to the “decrease probability” category (7.7 times more, p < 0.001) for both correct and incorrect diagnoses. We predicted, but did not observe, that the ratio of “decreases to increases” probability data would be higher for incorrect diagnoses compared to correct diagnoses (p = 0.41).

### RQ4: first year versus second year student performance

There were no differences observed for RQ3 between 1st and 2nd year medical students.

## Discussion

We provided deliberate practice and formative feedback for diagnostic clinical reasoning to pre-clerkship medical students starting in the first month of first year medical school. We observed and analyzed students’ CR performance for the following activities: building a Ddx, performing DxJ, ordering investigations, and selecting a final dx. We made five important observations. First, students scored: well on constructing the Ddx and choosing the correct diagnosis; moderately on ordering investigations; and poorly with diagnostic justification. Second, after diagnosing 10–11 cases with formative feedback, performance on DxJ increased by 40%; the other scores remained unchanged. Third, clinical data was rarely assigned as “decreasing probability” for diagnoses, even when the diagnoses were incorrect. Fourth, DxJ scores were lower for physical exam data than for history and investigation data. Fifth, second year students performed better than first year students on most measures; notably however, they did *not* outperform first year students on analyzing data from the investigations section nor on identifying the correct diagnosis.

DxJ skills are crucial for medical students to develop before they are ready for clinical practice [25]. Once a Ddx has been created, the process of DxJ provides the structure and evidence to demonstrate that both the correct diagnosis is indeed present and that all the incorrect diagnoses within the Ddx are concurrently absent. We believe that DxJ is the most important part of CR; the *absence* of DxJ is no better than assuming or guessing. If DxJ is not being performed, then what process *is* being used to determine the probabilities of the diagnoses within the Ddx?

Overall, the current findings suggest that DxJ is a skill that is difficult to learn and requires a lot of practice or experience to develop. The low DxJ scores we observed in pre-clerkship students accord with previous studies reporting that students’ DxJ scores were lower than expected *despite* exhibiting high final diagnostic accuracy [25, 28, 38]. One study showed that absences or major deficiencies in DxJ were observed in up to 48% of third year students [26].

Our study showed that DxJ scores improved 40% with one year of deliberate practice. This suggests that DxJ is indeed a skill that can be developed and improved with formative feedback. This observation supports Hayden et al., [38] who showed a dose-response relationship of increased DxJ scores with increased attendance of CR simulation. However, 1 year of practice is likely inadequate, as scores remained low (4.4 out of 10) after 1 year of training.

Within DxJ processes, we predicted and observed that students assigned more data as “increases” than “decreases” probability to their Ddx. However, contrary to our prediction, we did not observe more data assigned as “decreases” probability for the incorrect diagnoses compared to correct diagnoses. This finding complements previous studies reporting that novices neglected to use pertinent negative information [33–36]. Another finding within DxJ processes that is important was that scores for physical exam data analysis were lower than for history or investigations data; to our knowledge, this observation has not been previously reported. It is possible that inexperienced students tend to ignore or under-appreciate data from the physical exam and similarly, data that decreases the probability of diagnoses. It is also possible that current methods of instruction do not sufficiently train students to appropriately analyze these clinical data. These findings should receive attention in future CR training methods; we have upgraded our curriculum accordingly to explicitly highlight this feedback to students based on these findings.

We observed that scores for analyzing investigation results and identifying the final diagnosis were not different between 1st and 2nd year students; this observation could suggest that these metrics are invalid or ineffective methods of assessing CR. However, other publications have similarly concluded that final diagnostic accuracy is an insensitive CR assessment tool because students can get to the right diagnosis even if they score low on CR processes [21, 25, 28]. DxJ is likely a better indicator of the quality of underlying reasoning processes; when DxJ is incorporated into assessment of CR, it has been observed to be the most predictive of graduate competency exam performance, have the highest item discrimination and increases assessment reliability [26].

Strengths of this study include the large sample size, the large volume of data collected, and the highly organized storage structure of the data, making it easily analyzed at scale. The absence of multiple assessors of performance eliminates inter-observer variability since all scores were generated from a single unique scorecard for each case. We performed longitudinal data collection with 10–11 data collection events over a period of 6 to 10 months which we believe to be superior to a single assessment event. Furthermore, our data provides initial validity evidence based on observations that (1) second year students consistently outperformed first year students on most measures; (2) performance improved with practice; and (3) we replicated patterns found in previous studies on DxJ.

Limitations of the study include the generalizability of the data, the study design, and the creation of the scorecard. Generalizability was limited because all data were collected at a single site and on a limited number of cases. The study design was observational and does not allow us to draw causal conclusions. Lastly, given that this was the first year of deployment of this curriculum, we had two, but only sometimes three experts to review the scorecards of each case. We expect to discover and correct minor scorecard errors with increased expert review of the data in the scorecards.

Future research will extend our observations over 2 years and 20 cases for the same cohorts of learners. We are currently collecting data for a multi-centered project. We have upgraded our software to collect data to not only identify when and where the misdiagnoses occur, but to also inform why these misdiagnoses are occurring; this analysis will be included in our upcoming multi-site project.

In conclusion, pre -clerkship 1st and 2nd year medical students completed 10 and 11 deliberate practice CR cases respectively with formative feedback over one year in this single site study. Students scored particularly low for diagnostic justification processes, especially related to physical exam data, and assigning data as “decreases” probability. We did observe, however, that diagnostic justification scores improved by 40% within 1 year of deliberate practice. Diagnostic justification is a key component of CR and deliberate practice of CR starting in pre-clerkship students has now been shown to be a feasible and effective strategy to improve diagnostic CR skills.

### Electronic supplementary material

Below is the link to the electronic supplementary material.

Supplementary Material 1

## Data Availability

The datasets used and/or analysed during the current study available from the corresponding author on reasonable request.
