# Supplementary material for: Deliberate practice of diagnostic clinical reasoning reveals low performance and improvement of diagnostic justification in pre-clerkship students
Source: BMC Med Educ. 2023 Sep 21;23:684. doi: 10.1186/s12909-023-04541-5 (PMC10515060; doi:10.1186/s12909-023-04541-5)

**Supplementary Material A. Examples of the TeachingMedicine.com scorecard.**

Figure S1: Example of clinical information students could select from the case history.


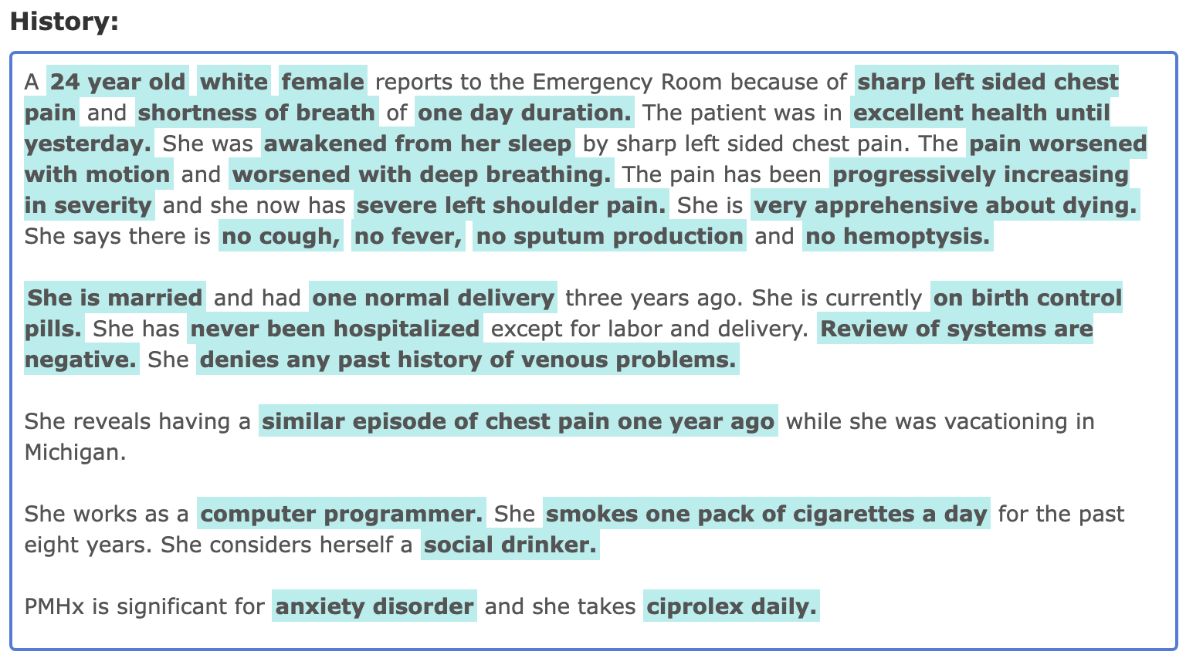


Figure S2: Example of clinical information students could select from the case physical exam.


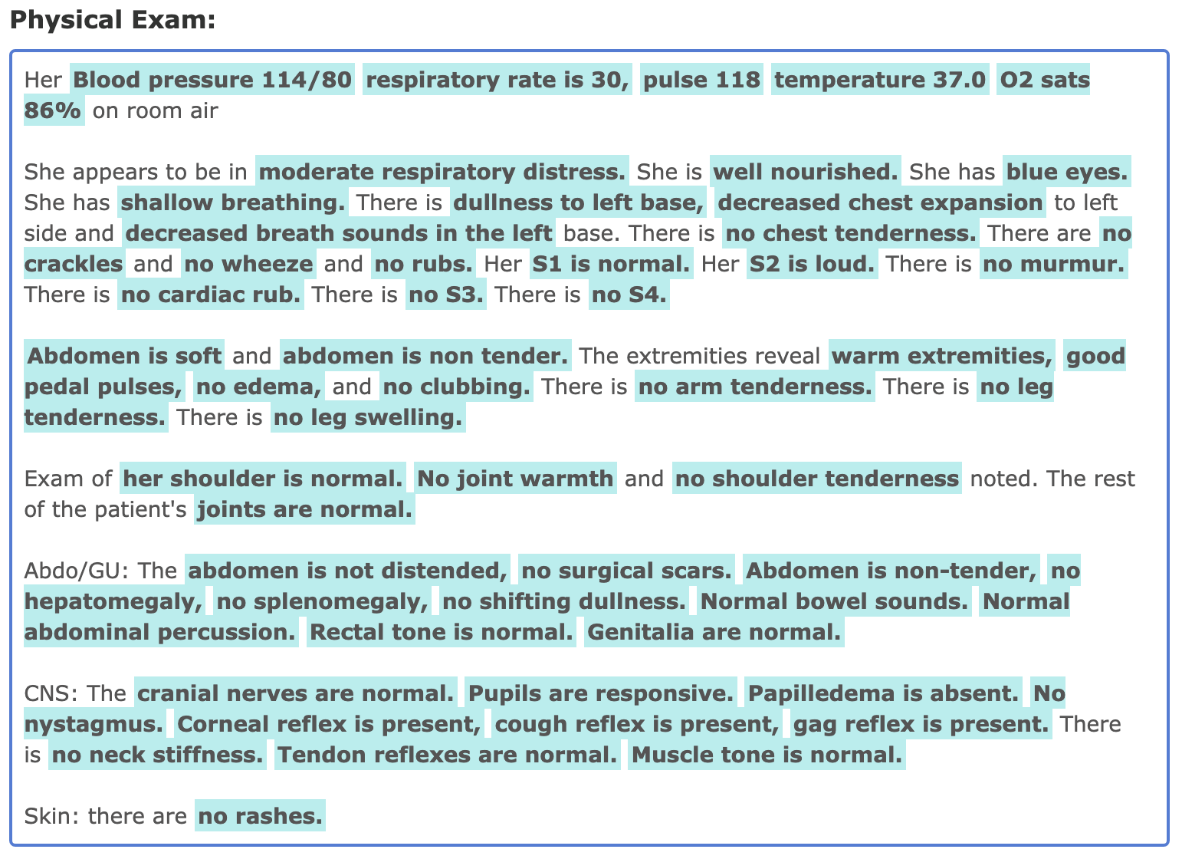


Figure S3. Scorecard for building the differential diagnosis (Ddx).


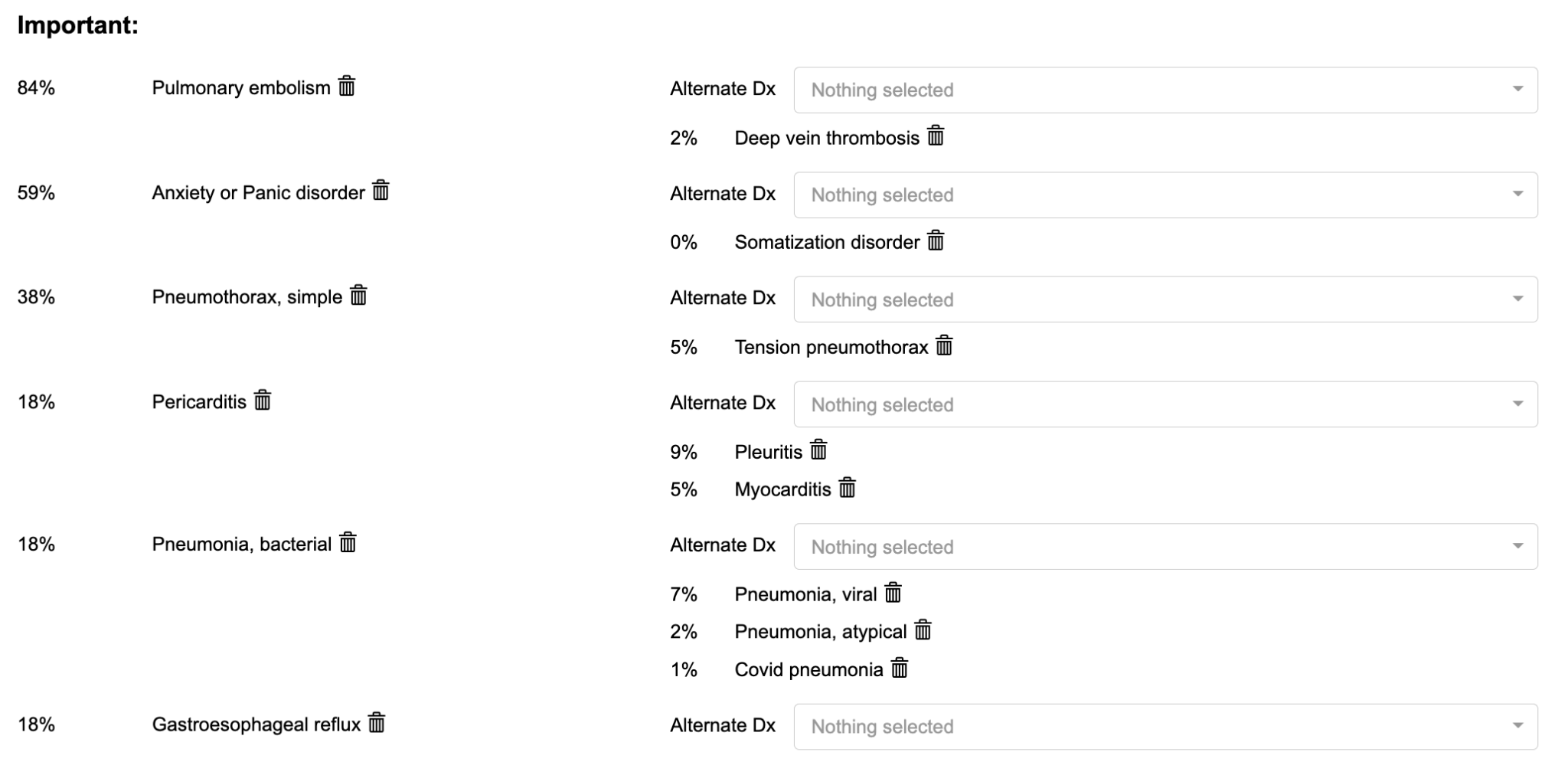


Figure S4. Scorecard for Diagnostic Justification.


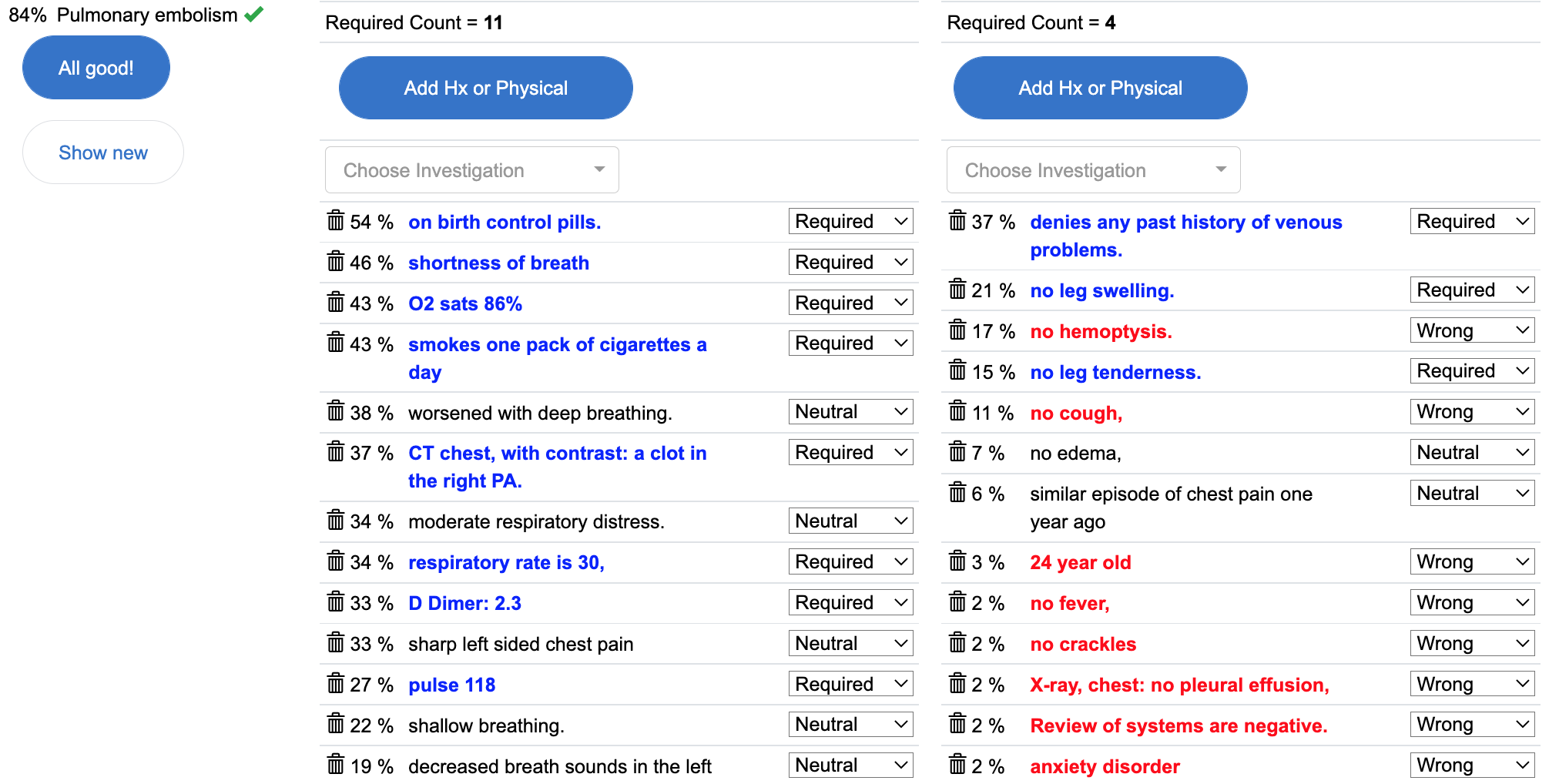


Figure S5. Scorecard for Investigations, consisting of a: Required Investigations; b: Inappropriate Investigations, and c: Conditional scoring based on Ddx.


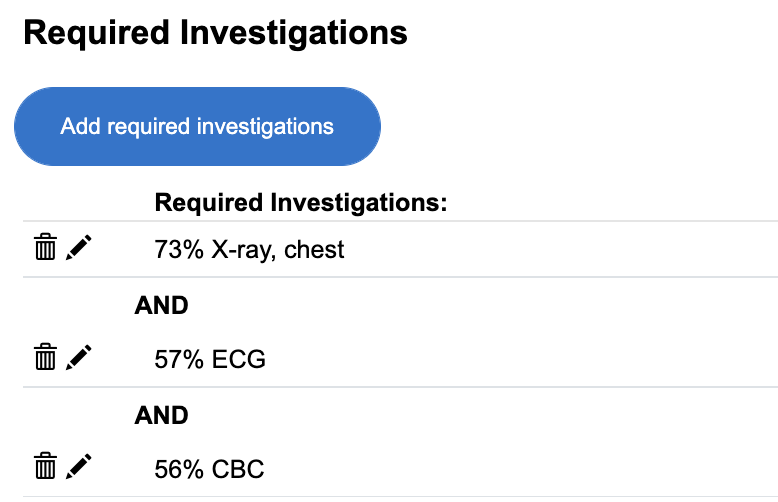


S5a

S5b


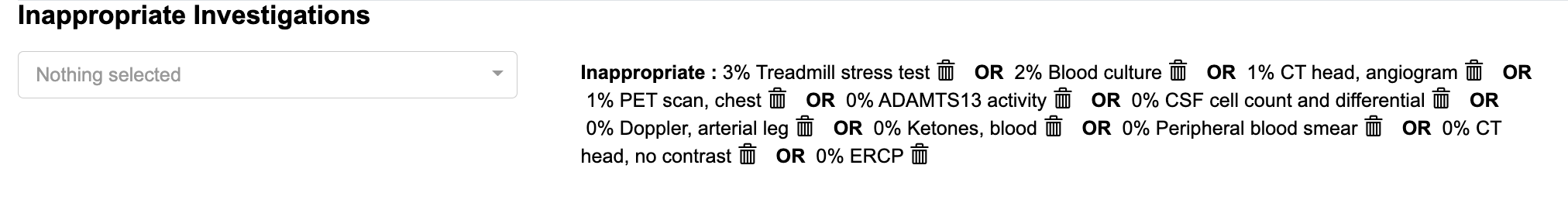


S5c


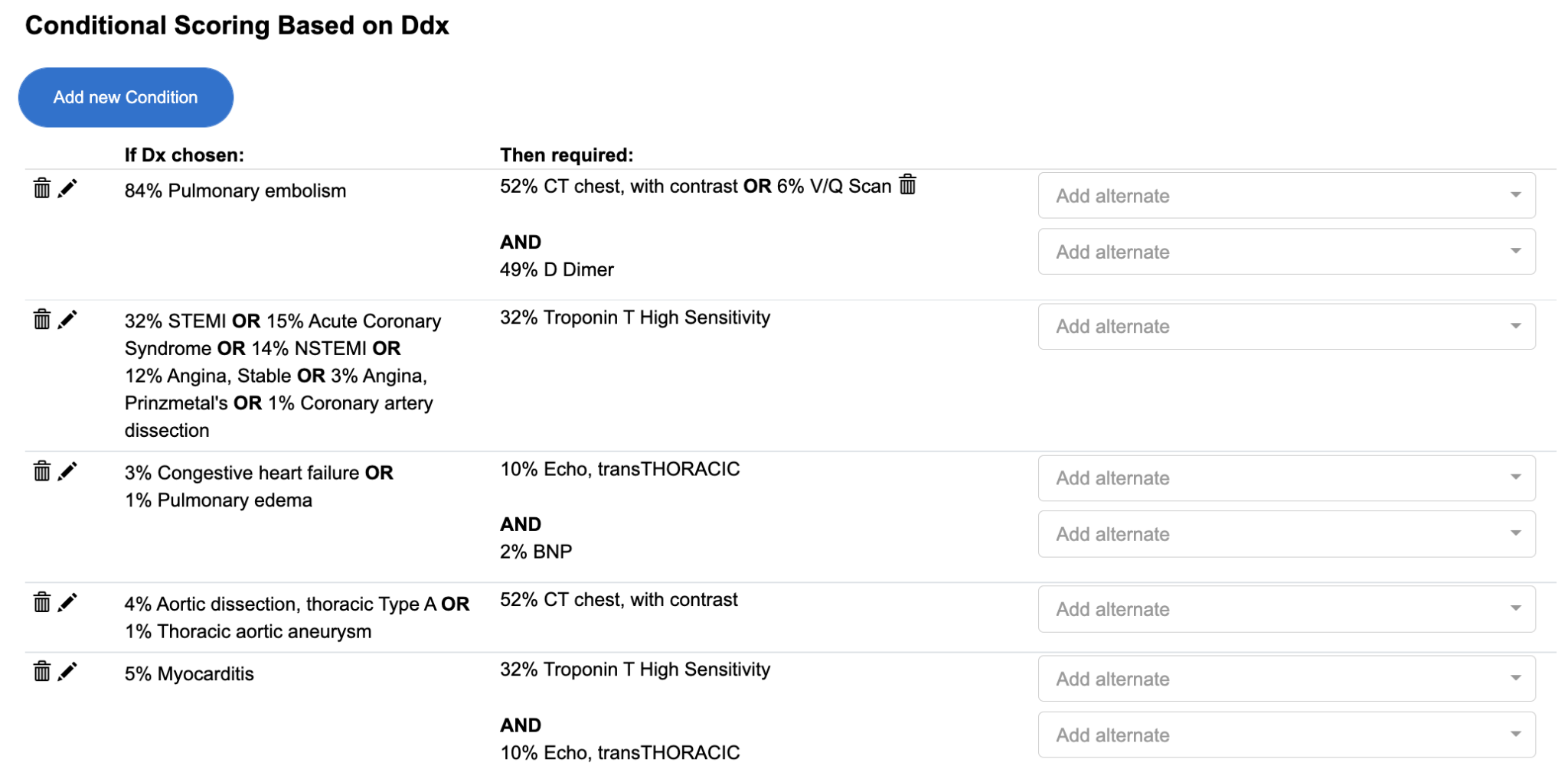

Supplement: Supplementary file 1 — Supplementary Material 1 [file 12909_2023_4541_MOESM1_ESM.docx]
